# Repair of a three-way congenital bronchoesophageal fistula in an adult patient: a case report

**DOI:** 10.1186/s12876-021-02033-8

**Published:** 2021-12-03

**Authors:** Kun Fan, Shan Gao, Rui Gao, Shuo Li, Junke Fu, Guangjian Zhang

**Affiliations:** 1 Department of Thoracic Surgery, The First Affiliated Hospital of Xi’an Jiaotong University, 277# Yanta West Road, Xi’an, 710061 Shaanxi China; 2 Department of Nuclear Medicine, The First Affiliated Hospital of Xi’an Jiaotong University, 277# Yanta West Road, Xi’an, 710061 Shaanxi China

**Keywords:** Bronchoesophageal fistulas, Double-layer repair, Esophagus in situ

## Abstract

**Background:**

The incidence of congenital bronchoesophageal fistulas in adults is rare. Most fistulas discovered in adulthood are often small and can be repaired with a simple one-step method.

**Case presentation:**

A 46-year-old female patient complained of a 2-month history of chocking, coughing, and a 12 kg drop in weight. The bronchofiberscopy and gastroscopy showed a large fistula, which extended from the esophagus to the main bronchus on both sides, thus forming a special three-way channel which has never been reported. This case was challenging both to the anesthetists and surgeons. The patient was intubated with a sengstaken-blakemore tube, and then received segmental esophageal resection, anastomotic reconstruction, and double-flap repair with esophagus segment in situ.

**Conclusion:**

When the fistula in BEF is large or complicated, appropriate surgical methods should be meticulously designed according to the condition of the patient. The problem of anesthesia intubation should be solved first, to allow a smooth operation. Secondly, a double-layer repair of the airway fistula by using esophageal wall tissues as patch materials is proposed.

## Background

Tracheoesophageal fistula (TEF) or bronchoesophageal fistula (BEF) is an abnormal communication between the esophagus and the airway. Congenital TEF/BEF is characterized with severe symptoms, and is rarely diagnosed at adulthood [1]. Although congenital TEF/BEF is benign, if left untreated, it may lead to fatal complications. Therefore, timely surgical treatment should be performed following diagnosis [2]. We herein report an extremely rare case of an adult with three-way congenital BEF.

## Case presentation

**Fig. 1:**
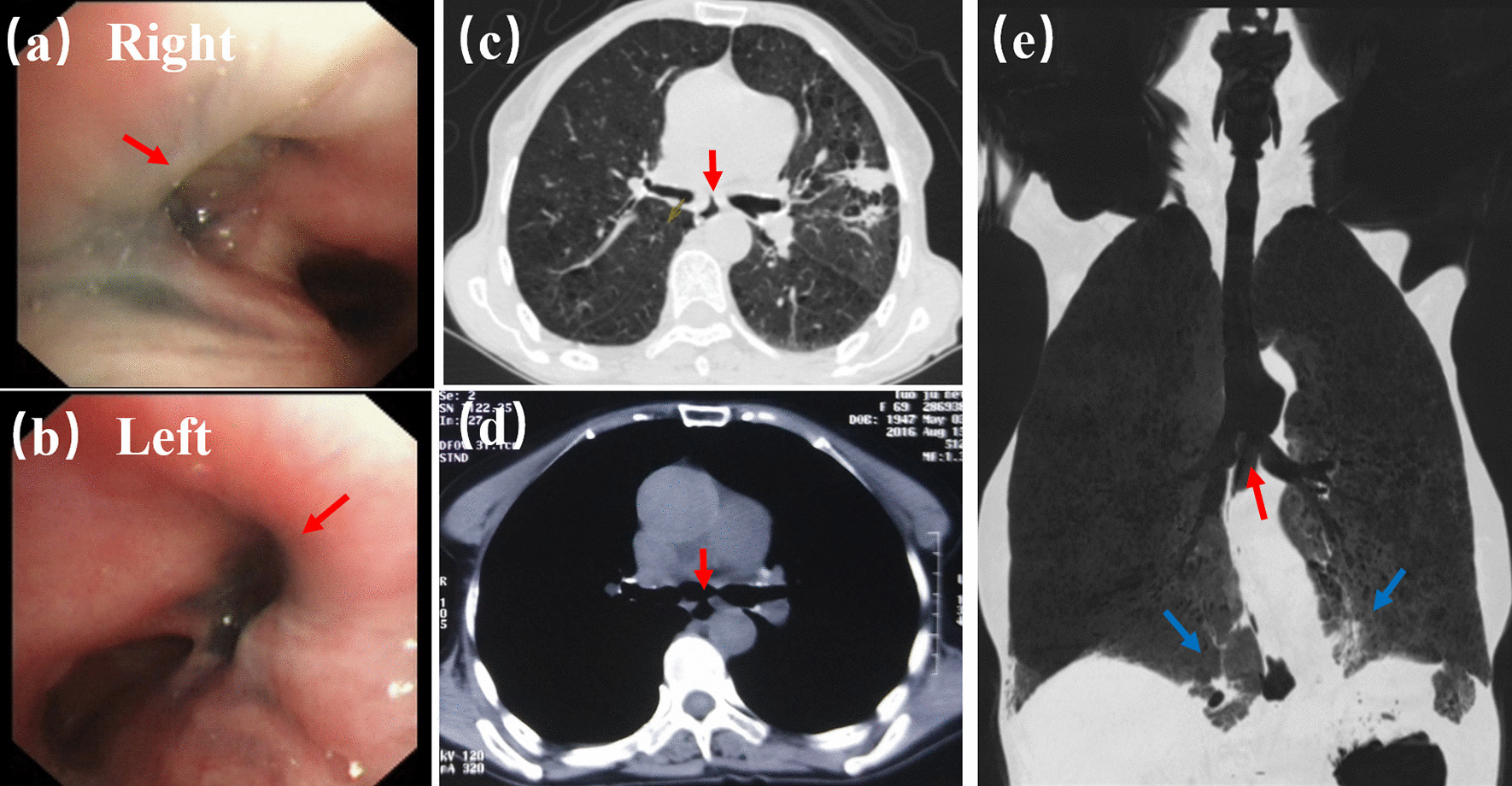
Preoperative imaging examination (red arrows indicate opening fistula, while blue ones indicate lung pneumonia). **a**, **b** Bronchofiberscopy showing a double-opening fistula in the posterior-interior wall of the trachea. Chest CT results demonstrating a three-way tracheoesophageal fistula (**c**, **d**) and pneumonia in the lower lung field (**e**)

A 46-year-old female patient was admitted to out hospital with a 2-month history of chocking, coughing, and a 12 kg drop in weight. The patient presented with no symptoms of fever, chest pain, difficulty breathing or history of esophageal tumors, inflammation, and trauma. Bronchofiberscopy revealed a double-opening fistula in the posterior-interior wall of the trachea (Fig. 1a, b). Gastroscopic examination also demonstrated a 2 cm defect in the frontier wall of the esophagus arising 27 cm from the incisor teeth. Chest CT confirmed three-way BEFs and pneumonia in the lower lung field (Fig. 1c–e).

She was put on supportive therapy with nutritional support, respiratory function training, and anti-infection treatment, all of which improved her physical condition allowing for surgical treatment. During preoperative anesthesia, a sengstaken-blackmore tube was inserted. We used the esophageal sac to block the esophageal fistula and form a complete airway circuit. In addition, the stomach sac was used to seal the cardia thereby prevent gastrobronchial reflux. Subsequently, ordinary tracheal intubation was performed to improve ventilation.

**Fig. 2:**
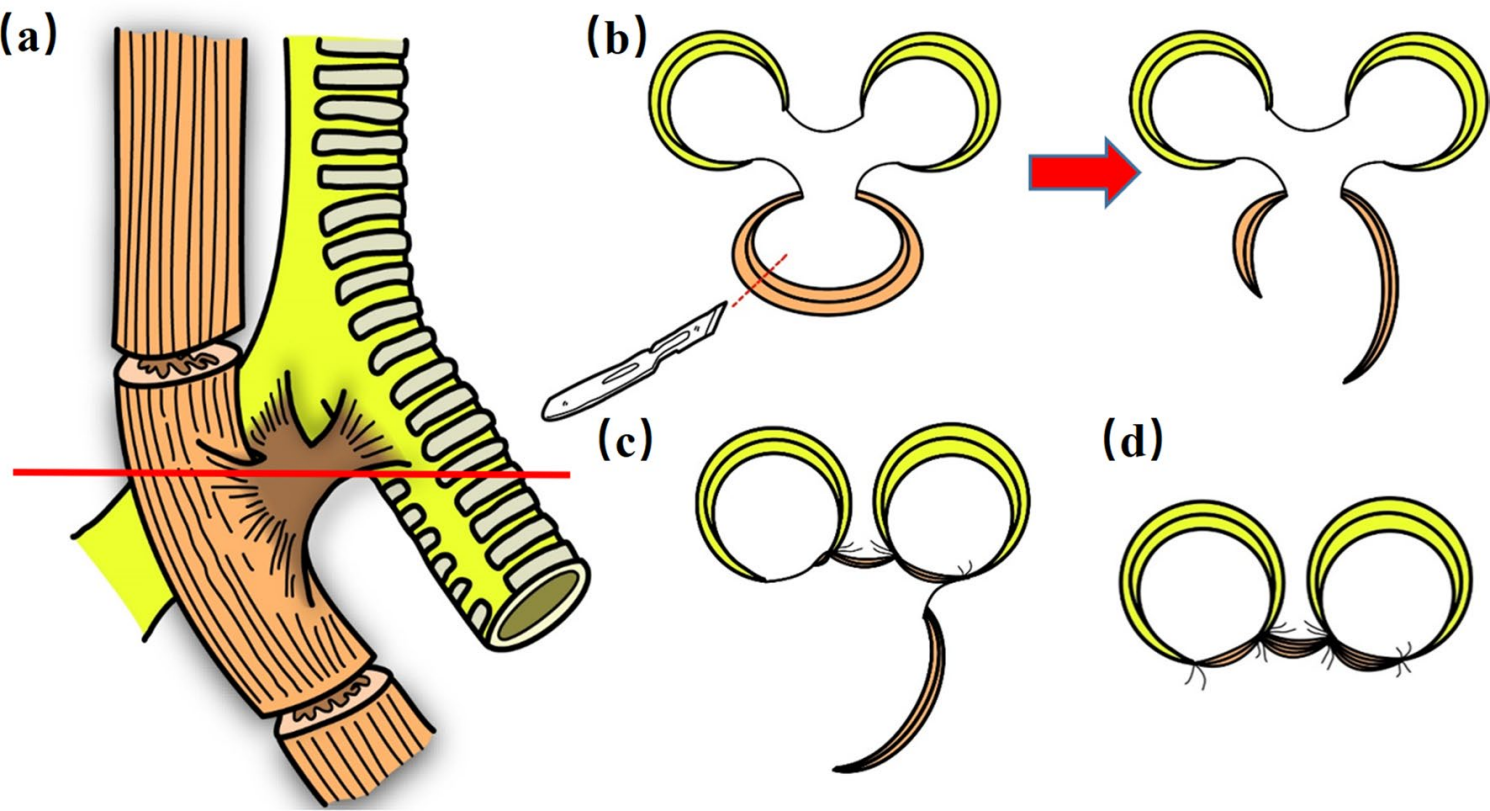
The use of esophageal patches to surgically repair the fistula (the red line represents the slice level of the next three pictures). **a** Cut the esophagus 2 cm from the top and bottom of the lesion. **b** Cut the remaining esophagus to form two flaps, one large and one small. **c**, **d** Repair the fistula with double esophageal flap

During surgery, we first created a tubular stomach through laparoscopy and performed jejunostomy to ensure adequate postoperative nutrition. Then, a left neck incision was made through which the tubular stomach was lifted towards the neck via the retrosternal passage. This allowed gastroesophageal anastomosis to be performed to rebuild the continuity of the digestive tract. Finally, thoracotomy was conducted through the right-side incision. The thoracic segment of the esophagus was mobilized above and below the fistula, approximately 2 cm away from the fusion of the trachea with the esophagus (Fig. 2a). The remaining esophagus around the fistula was cut from the middle 2/3 to form a short muscle flap and a long one (Fig. 2b). The short flap was first used to suture the edge of the corresponding fistula (Fig. 2c), covered with the long flap and finally fixed with sutures to form a double-layer repair (Fig. 2d).

**Fig. 3:**
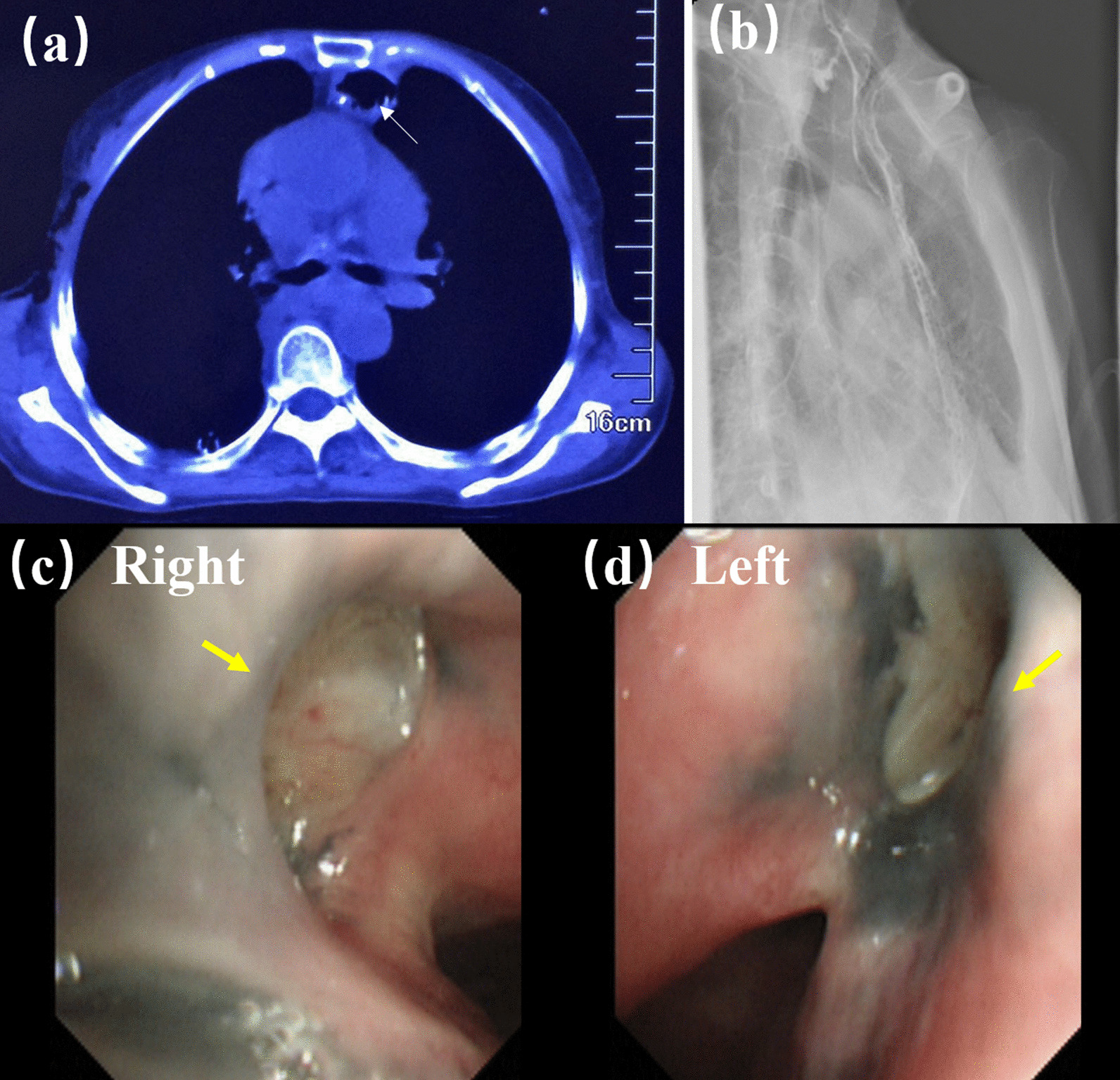
Postoperative imaging examination (yellow arrows indicate closured fistula). **a** CT results showing complete disappearance of the fistula (the white arrow points to the tubular stomach). **b** Barium esophagogram showing the absence of anastomotic leakage. **c**, **d** Bronchoscopy showing that the fistula was repaired well

The postoperative course was uneventful. A chest CT performed on the 5th day after surgery showed that the fistula had disappeared (Fig. 3a). Barium esophagogram conducted on the 11th day after surgery confirmed the absence of anastomotic leakage and the patient was allowed to drink water (Fig. 3b). Bronchoscopy was performed on the 12th day after surgery and the result showed that the fistula was repaired well (Fig. 3cd). Two years postoperatively, the patient showed satisfactory progress without any respiratory or swallowing problems.

## Discussion and conclusions

Congenital TEF/BEF was first reported by Negus early in 1929 [3]. The common embryonic origin of the respiratory tract and esophagus may cause abnormal congenital traffic between them, leading to the formation of TEF or BEF. TEF/BEF is commonly diagnosed in infants with esophageal atresia. In rare cases, its symptoms may be concealed leading to adulthood diagnosis. In this case, the lesions are often narrow, small and relatively easy to correct. However, fistula diagnosed in the present case was large and complicated. This was possibly because local mucosal folds acted as a valve and blocked the passage from the esophagus to the trachea. This may also explains why the patient had delayed symptoms and survived until adulthood.

Clinically, the repair of TEF/BEF is a complex and challenging surgical process which may be accompanied with complications and high risk of mortality. Nevertheless, surgical treatment often results in good long-term outcomes with 90% of all fistulas eventually closed [2]. Hence, surgery is recognized as the first-line treatment for fistulas. The choice of appropriate surgical procedure is dependent on several factors including the severity of symptoms, the size and location of the fistula, and the underlying patient conditions. Given to the particularity of the present case, it was impossible to remove both main bronchi around the fistula at once. Therefore, our aim was to remove the diseased esophagus, rebuild the continuity of the digestive tract and repair the bronchial fistula without removing it. Lobectomy was not performed because of the severe lung inflammation.

Proper anesthetization is critical before the operation. To prevent gastric distention and loss of ventilation volume, the cuff of the tracheal intubation should be placed under the fistula, or the dual-lumen endotracheal intubation should be used to provide unilateral lung ventilation and avoid the fistula [4]. However, bilateral bronchial defects found in the present case made it impossible to perform traditional intubation. Moreover, gastrotracheal reflux is more likely to occur given the high intra-abdominal pressure caused by laparoscopic pneumoperitoneum. To address challenges, we creatively employed the sengstaken-blakemore tube.

Since most of the reported congenital BEFs are simple small fistulas, one-step repair, including direct fistula closure or fistula resection with flaps of soft tissue/muscle interposed to reinforce the suture by surgical or endoscopic approach are often applied. However, the one-step repair is only recommended for small non-patulous fistulas [5]. For larger patulous fistulas, two-stage approach, including closure of the defects with esophageal or tracheal patches, followed by esophageal/tracheal resection and anastomotic reconstruction, have been advocated [6]. Moreover, double-patching repair with esophageal wall has been suggested for patients with huge (> 5 cm) or complicated fistulas. The segmental blood supply of the esophagus improves the long-term survival of the esophageal patch. Application of pedicle regional flaps or microvascular free flaps in the reconstruction of enlarged tracheoesophageal puncture sites is well-documented approach. In current case, the esophageal tissues around the lesion were utilized to form a “natural” membranous wall in situ to repair the fistula on the bronchus and the recovery was satisfactory. To our knowledge, this is the first report of a case of congenital BEF with three-way openings that was successfully treated using esophageal wall tissues as protective patches.

In conclusion, when the fistula in BEF is large or complicated, appropriate surgical methods should be meticulously designed according to the condition of the patient. The problem of anesthesia intubation should be solved first, to allow a smooth operation. Secondly, a double-layer repair of the airway fistula by using esophageal wall tissues as patch materials is proposed.

## Data Availability

The datasets supporting the conclusions of this article are included in the article.

## Abbreviations

BEF: Bronchoesophageal fistulas
TEF: Tracheoesophageal fistula

## References

[1] Zhang BS, Zhou NK, Yu CH (2011). Congenital bronchoesophageal fistula in adults. World J Gastroenterol.

[2] Bibas BJ, Cardoso PFG, Minamoto H (2018). Surgery for intrathoracic tracheoesophageal and bronchoesophageal fistula. Ann Transl Med.

[3] Lansden FT, Falor WH (1960). Congenital esophagorespiratory fistula in the adult. J Thorac Cardiovasc Surg.

[4] Ranjan RV, Ramachandran TR, Veliath DG (2012). Anesthetic management of congenital broncho-esophageal fistula in an adult. Ann Card Anaesth.

[5] Azoulay D, Regnard JF, Magdeleinat P (1992). Congenital respiratory-esophageal fistula in the adult: report of nine cases and review of the literature. J Thorac Cardiovasc Surg.

[6] Saxena P, Tam R (2006). Late manifestation of a large congenital tracheoesophageal fistula in an adult. Tex Heart Inst J.

